# Molecularly Imprinted Electrochemical Sensor for the Ultrasensitive and Selective Detection of Venetoclax

**DOI:** 10.1021/acsomega.4c03819

**Published:** 2024-08-08

**Authors:** Ece Ozkan, Esen Bellur Atici, Sibel A. Ozkan

**Affiliations:** † Ankara Medipol University, Faculty of Pharmacy, Department of Analytical Chemistry, 06239 Altındağ, Ankara, Türkiye; ‡ DEVA Holding A.S., R&D Center, Tekirdağ 59600, Türkiye; § Ankara University, Faculty of Pharmacy, Department of Analytical Chemistry, Ankara 06100, Türkiye

## Abstract

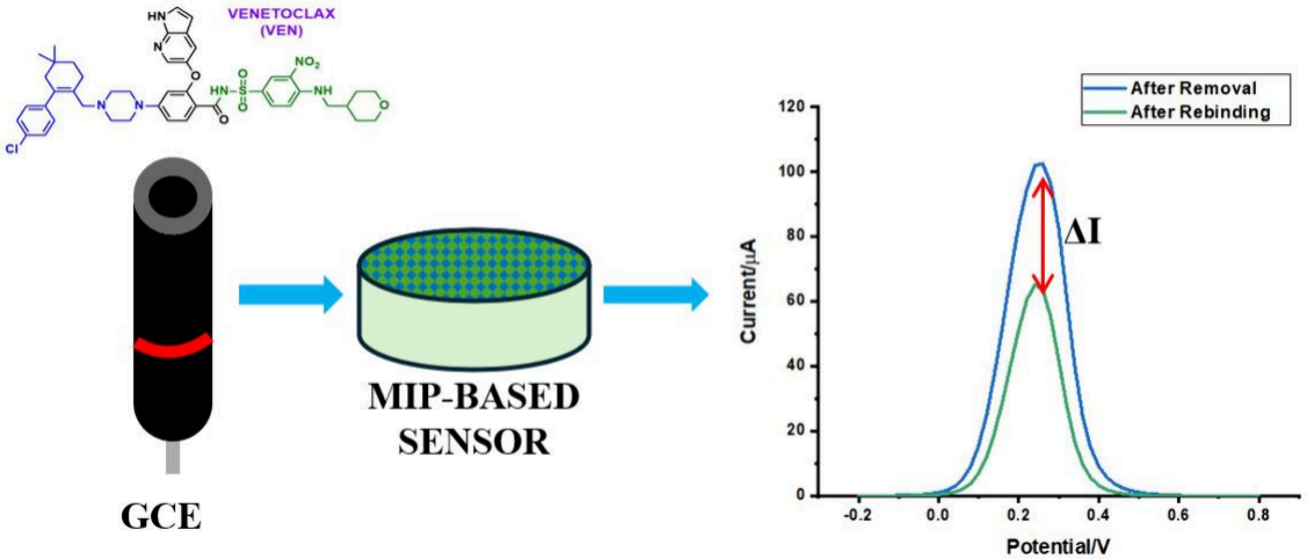

In this study, a new electropolymerized molecularly imprinted polymer (MIP) film was synthesized on a glassy carbon electrode (GCE) by a photopolymerization (PP) method using acrylamide (AA) as a functional monomer and venetoclax (VEN) as a template molecule. Optimization steps of the MIP film were performed using ferrocyanide/ferricyanide [Fe(CN)_6_]^3–/4–^ as a redox probe. Removal and rebinding of the template molecule were investigated by differential pulse voltammetry (DPV) and electrochemical impedance spectroscopy (EIS). The analytical performance of PP-AA@MIP-GCE was evaluated by comparing the DPV response of MIP with that of nonimprinted polymer (NIP). The limit of detection (LOD) and limit of quantification (LOQ) for DPV determination of VEN on PP-AA@MIP-GCE were 0.016 and 0.055 pM, respectively, and the linearity range was found to be between 0.1 and 1.0 pM. The applicability and legitimacy of the constructed sensor were confirmed through its utilization on synthetic human serum. The selectivity of the sensor was demonstrated using molecules with structures similar to that of VEN and/or drug substances such as ibrutinib and azacitidine, which could potentially be used in combination with VEN. The developed PP-AA@MIP-GCE sensor exhibited high sensitivity and selectivity for VEN and is the first reported method for DPV determination of VEN.

## 1 Introduction

VEN (2-((1*H*-pyrrolo[2,3-*b*]pyridin-5-yl)oxy)-4-(4-((4′-chloro-5,5-dimethyl-3,4,5,6-tetrahydro-[1,1′-biphenyl]-2-yl)methyl)piperazin-1-yl)-*N*-((3-nitro-4-(((tetrahydro-2*H*-pyran-4-yl)methyl)amino)phenyl)sulfonyl)benzamide, C_45_H_50_ClN_7_O_7_S, (868.44 g/mol) (Table 3) is an oral selective inhibitor of B-cell lymphoma-2 (Bcl-2).^1^ It is an important drug for the treatment of chronic lymphocytic leukemia (CLL) and acute myeloid leukemia (AML).^2,3^ Patients with relapsed and resistant CLL were given approval to treat with VEN in 2016 by the European Medicines Agency (EMA) and the US Food and Drug Administration (FDA).^4^ VEN has demonstrated effectiveness both when used alone and in conjunction with other treatments in individuals suffering from hematologic malignancies. Efficacy and tolerability are considered when determining the appropriate treatment. Therefore, the appropriate dose of VEN is of paramount importance.^1^ After a single dose, the peak plasma concentration of VEN is reached 6–8 h later, and in CLL patients, the terminal elimination half-life (t1/2) was roughly 19 h.^2^ In drug–drug interaction studies (ketoconazole and rifampin), VEN was found to be a cytochrome P450 (CYP)3A substrate and significant changes in VEN concentrations were observed.^5,6^ Weak inhibition of cytochrome P450 (CYP) 2C9 by VEN has been demonstrated in in vitro studies. In addition, VEN is a P-glycoprotein (P-gp) inhibitor and is highly protein-bound (99%).^7^

Electrochemical techniques have become the preferred methods in analytical applications because they are simple, fast, inexpensive, and can achieve very low LOD. It is necessary to strengthen the electrochemical sensors’ performance to increase the sensitivity and selectivity of electrochemical techniques. MIP can separate target molecules from complex sample matrices with high sensitivity, selectivity and superior detection capability.^8^ MIPs are formed by polymerizing target molecules with the appropriate functional monomer. Cross-linkers and initiators can also be used during polymerization. This formation creates specific recognition sites with shape and functional groups complementary to the target analyte, enabling MIPs to recognize template molecules. MIPs are widely used due to their advantages of easy preparation, high affinity, selectivity, sensitivity and stability.^9−11^

AA (2-propenamide, C_3_H_5_NO) enhances the hydrogen bonding effects to strengthen the binding relationship in the template-monomer complex. AA copolymers are water-soluble, which increases the likelihood of monomer binding and potential denaturation. AA-based molecularly imprinted polymers (MIPs) have shown promise.^12,13^

There is a limited amount of literature on VEN analysis. The plasma concentration of VEN was determined using high-performance liquid chromatography (HPLC) with a linearity range of 0.25–10 μg/mL and LOQ of 0.10 μg/mL. The recovery was >97.2% in a plasma sample taken from a patient administered 50 mg of VEN after breakfast.^1^ The VEN and its decomposition products were detached using reversed-phase HPLC with a linearity range of 5.00–0.02 μg/mL. The LOD and LOQ values were 0.075 μg/mL and 0.188 μg/mL, respectively. The recoveries ranged from 98.66% to 101.65%.^4^ In another study, VEN is determined in human plasma using liquid chromatography-tandem mass spectrometry (LC-ESI-MS/MS) with a linear concentration range of 5.0–5000.0 pg/mL. The LOD and LOQ values were 5 pg/mL and 50 pg/mL, respectively. The mean recovery of VEN was observed to be 90.3% and 92.5%, respectively.^14^ Furthermore, a reverse-phase HPLC method was developed to determine the concentrations of VEN and obinutuzumab in both bulk and pharmaceutical forms. The linearity range for VEN was 3–45 μg/mL, with LOD of 0.03 μg/mL and LOQ of 0.0075 μg/mL. The recoveries were within the acceptable range of 98% – 102%.^15^ In a phase 1 trial, the impact of VEN on the pharmacokinetics of warfarin was examined in healthy participants, it was observed that the maximum plasma concentration of R- and S-warfarin and the area under the curve to infinite time values increased by approximately 18 to 28%.^7^ Additionally, a study was carried out on the impact of azithromycin on VEN pharmacokinetics in healthy volunteers. According to the study, the combination of VEN and azithromycin resulted in a 25% decrease in maximum concentration and a 35% decrease in area under the curve to infinity, compared to using VEN alone.^16^

A novel electrochemical sensor was developed to quantify VEN using MIP-based copolymerization of AA as the functional monomer. AA is the functional monomer that interacts with VEN and has the primary activity in polymerization, while 2-Hydroxyethyl methacrylate (HEMA) is the basic monomer involved in the formation of the polymerization chain. The sensor possessed high sensitivity, good selectivity, and simplicity. The performance of the PP-AA@MIP-GCE and the electrochemical behavior of VEN were investigated. The MIP-based sensor was successfully used to quantify VEN in commercial human serum samples by the DPV. This work, describes the first MIP-based electrochemical sensor to evaluate VEN in biological specimens, exhibiting superior specificity, exceptional sensitivity, stability, and precision.

## 2 Experimental Section

### 2.1 Reagents and Chemicals

VEN and other active drug substances (≥99.5%), provided by DEVA Holding A.Ş. (İstanbul, Türkiye), were utilized directly in the study. A stock solution of VEN at a concentration of 1.0 mM was prepared using dimethyl sulfoxide (DMSO, ≥ 99.5%, Merck). Potassium ferricyanide (K_4_[Fe(CN)_6_], ≥ 99.0%), and potassium ferrocyanide (K_3_[Fe(CN)_6_], ≥ 98.5%) were purchased from Merck. The following reagents were obtained from Sigma-Aldrich: methanol (MeOH, 99.8%), 2-hydroxy-2-methylpropiophenone (>97.0%), AA (≥99.0%), sodium sulfate (>99%), sodium chloride (>99%), dopamine (DOP, ≥ 97.5%), ethylene glycol dimethacrylate (EGDMA, > 98.0%), sodium hydroxide (>97%), acetic acid (AcOH, ≥ 99.8%), sodium acetate trihydrate (>99%), potassium chloride (>99%), 2-hydroxyethyl methacrylate (HEMA, ≥ 99.0%), and drug-free human serum (from male AB originated in the USA). To maintain their freshness, every chemical and stock solution utilized in the study was kept in a refrigerator at around 4 °C.

### 2.2 Instruments

Ivium Compactstat potentiostats with IviumSoft software were used for CV, DPV and EIS measurements (Netherlands). Ivium Compactstat potentiostats contain three electrodes consisting of a working electrode of GCE (3 mm diameter), a counter electrode of Pt wire, and a reference electrode of saturated Ag/AgCl (3 M KCl) in the electrochemical cell. UV lamp (100 W, 365 nm) was used to perform PP. A Thermo-Shaker (Biosan TS-100) was used to remove and rebind the template. The surface morphology of the films was investigated using a scanning electron microscope (SEM, TESCAN GAIA 3, Brno-Kohoutovice, Czech Republic).

### 2.3 Preparation of the MIP- and NIP-Based Electrochemical Sensors

MIPs for sensing were prepared using the PP method, as described by Ozkan et al.^17^ First, the prepolymer complex was prepared in distilled water by mixing 20 μL 1.0 mM AA and 20 μL 1.0 mM VEN in a 1:1 ratio (v/v). HEMA (20 μL) and EGDMA (100 μL) were added to the AA/VEN complex. The homogeneous solution was then mixed on an orbital shaker for 30 s at ∼25 °C. The PP solution of the NIP contains all the same ingredients except for the template molecule VEN. After adding 2 μL of 2-hydroxy-2-methylpropiophenone as an initiator, 0.5 μL of the PP solution was applied to the GCE surface. Polymerization was performed by exposure to a UV lamp for 7 min, followed by 7 min at room temperature.

After creating a polymeric film on the surface using this process, the next step was to remove the template molecule to obtain cavities particular to VEN. The GCE was immersed in a 15 M acetic acid solution of VEN for 10 min on a shaker (650 rpm, 25 °C) to remove the template molecule. Subsequently, the GCE was incubated for 7 min under optimal conditions to rebind different concentrations of the target molecule VEN. The performance verification process for PP-AA@MIP-GCE was carried out at each step using the NIP-based GCE and prepared according to the same protocol. All measurements (DPV, CV and EIS) were carried out indirectly by utilizing the electrochemical response of a 5 mM [Fe(CN)_6_]^3–/4–^ solution prepared in 100 mM KCl.

### 2.4 Preparation of Commercial Human Serum Sample

To prepare a 0.1 mM serum standard solution, 3600 μL of a commercial serum sample stored at −20 °C was combined with 5400 μL of acetonitrile and 1000 μL of VEN stock solution. The tubes were sonicated for 15 min and centrifuged at 5000 rpm, at 25 °C for 20 min. The resulting supernatant was transmitted to an electrochemical cell for measurements. Recovery studies were conducted using the standard addition method with DPV.

## 3 Results and Discussion

### 3.1 Characterization of PP-AA@MIP-GCE

**Figure 1:**
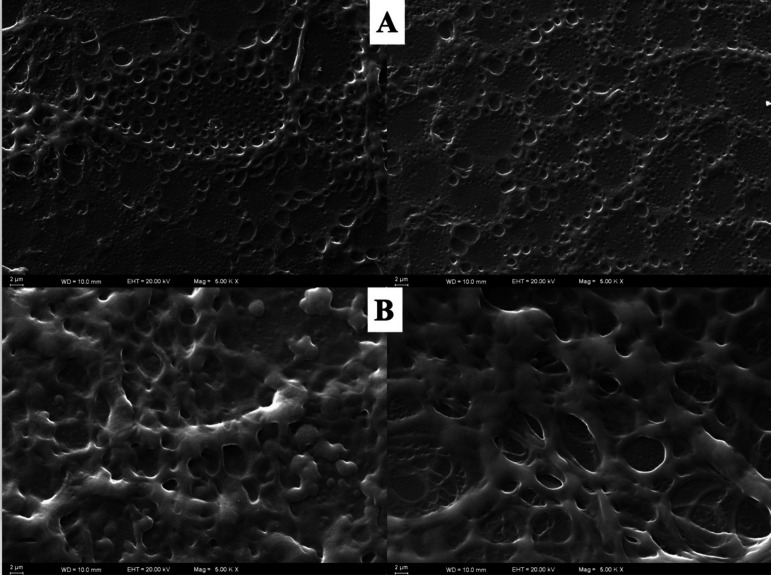
Characterization of the electrode surface. SEM images of PP-AA@MIP-GCE sensor (A) MIP and (B) NIP surfaces.

SEM images were analyzed to investigate the sensor surface and to compare the MIP-based surface with the NIP-based surface. The SEM images illustrate the structural differences between the surfaces of PP-AA@MIP-GCE (Figure 1A) and PP-AA@NIP-GCE (Figure 1B) sensors. Indicating the existence of particular binding sites created by the MIPs, the rough and porous texture seen on the MIP surfaces is a direct consequence of the modification process. In contrast, the NIP-based sensor’s surface is smooth with few irregularities. These results are consistent with the foreseen behavior of NIPs, that do not possess the special attachment sites characteristic of MIPs.

### 3.2 Electrochemical Characterization of PP-AA@MIP-GCE

**Figure 2:**
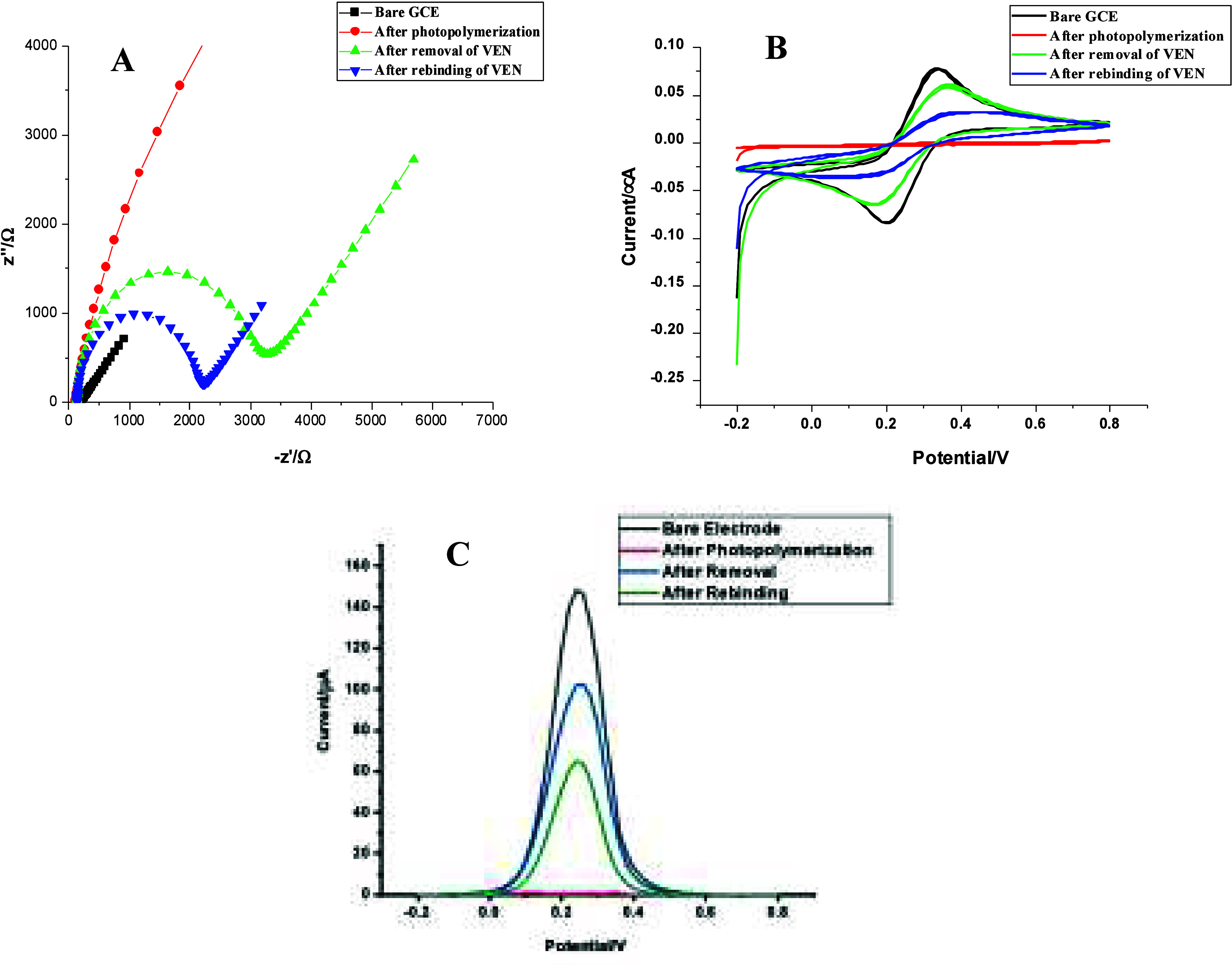
(A) Nyquist plots, (B) CVs and (C) DP voltammograms of bare GCE, PP-AA@MIP-GCE after PP, PP-AA@MIP-GCE after removal of VEN, and PP-AA@MIP-GCE after rebinding of VEN in a 5 mM [Fe(CN)_6_]^3–/4–^ solution. MIP/GCE sensor preparation. (Conditions for EIS: minimum frequency: 0.1 Hz, maximum frequency: 100,000 Hz, Eac: 0.01 V; for CV: potential scan range:–0.2 V to+0.8 V, scan rate: 0.05 V/s, step potential: 0.01 V; for DPV: potential scan range, – 0.2 V to+0.8 V; scan rate, 50 mV/s; step potential 8 mV; modulation amplitude 50 mV; modulation time 0.05 s, interval time 0.5 s.)

The electrochemical characterization of the VEN was conducted by comparing CV and EIS methods of the PP-AA@MIP-GCE sensor in a 5 mM [Fe(CN)_6_]^3–/4–^ solution. Figure 2A shows the changes in the impedance spectrum. The bare GC sensor exhibited low charge transfer resistance (R_ct_) values of 120 Ω, whereas, after polymerization, this value increased to 7528 Ω. After removal, the R_ct_ decreased to 1017 Ω. However, upon rebinding of VEN to the sensor surface, there was a significant increase in the R_ct_ value to 1584 Ω.

The electrochemical features of the PP-AA@MIP-GCE sensor were assessed using CV with a 5 mM [Fe(CN)_6_]^3–/4–^ redox probe. The assessment was conducted prior to and following the PP process, subsequent to the exclusion of the template molecule VEN, and after incubation with the template molecule VEN, as illustrated in Figure 2B. The GCE produced the highest CV peak current for [Fe(CN)_6_]^3–/4–^ However, after PP, the CV peak current decreased significantly because of constraint of electron transfer caused by polymer formation. Removing the VEN significantly increased CV peak current as the template molecule cavities were structured. Upon rebinding the VEN, the CV peak current decreased again to a lower level due to the reoccupation of the imprinted cavity. In addition, the DPV characterization of each step of the GCE is given in Figure 2C.

### 3.3 Optimization of PP-AA@MIP-GCE Parameters

**Figure 3:**
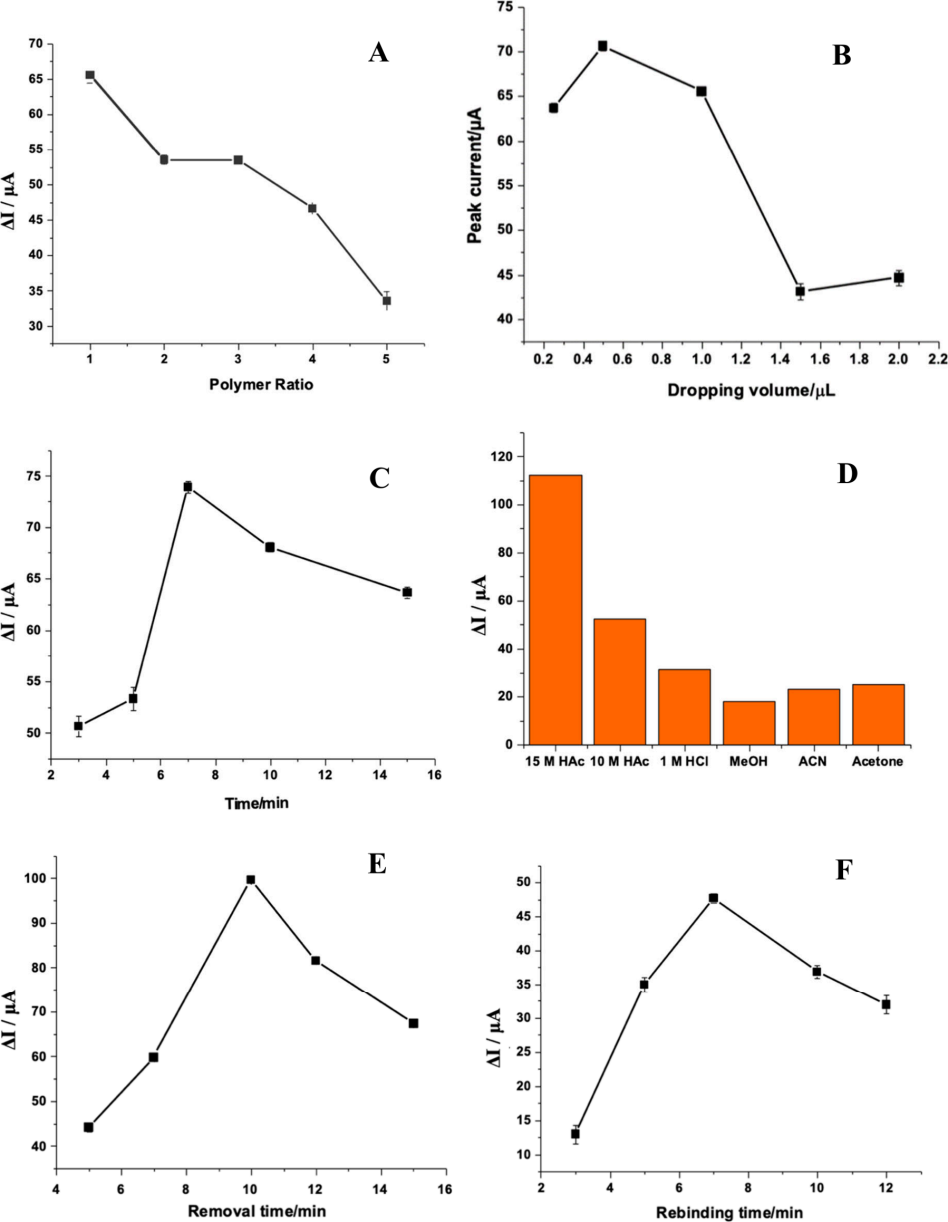
Plot of ΔI values versus (A) different AA/VEN ratios, (B) dropping volume, (C) polymerization time, (D) removal solutions, (E) removal time, and (F) rebinding time in a 5 mM [Fe(CN)_6_]^3–/4–^ solution (scan rate was 50 mV/s for DPV method) (*n* = 3).

Optimization studies were conducted to determine the optimal MIP-based sensor for the determination of VEN (Figure 3).

#### 3.3.1 Effect of Monomer/Template Ratio

If the functional monomer:template ratio is lower than it should be, functional groups cannot take part effectively in the resulting polymeric structure, and problems occur in the formation of selective cavities. If the ratio is too high, large amounts of functional monomers will be placed irregularly in the polymeric structure, not forming selective cavities. For these reasons, peak current values (ΔI) recorded after removal and after polymerization were taken into consideration, and the ratio of 3:1, where both effective polymerization and removal, which enables the formation of selective cavities, took place, the ΔI value was the highest, was preferred.

The purpose of this study was to examine the impact of different ratios of monomer to target molecule on the polymer. Specifically, we tested ratios of 1:1, 2:1, 3:1, 4:1, and 5:1 (v/v). The optimal monomer/target molecule (AA/VEN) ratio for the preparation of PP-AA@MIP-GCE sensor was found to be 1:1 (v/v) (Figure 3A).

#### 3.3.2 Dropping Volume for Polymerization Solution

To optimize the volume of the polymerization solution, 0.25 μL, 0.5 μL, 1.0 μL, 1.5 μL, and 2.0 μL were dropped onto the electrode surface. The highest peak current difference was obtained after polymerization and removal of the template molecule using 0.5 μL of the polymerization solution (Figure 3B).

#### 3.3.3 Polymerization Time

The duration of exposure to UV light for polymerization, from dropping the polymerization solution on the electrode surface up until obtaining the best MIP film formation, was tested between 3 and 15 min at five different points. The results indicate that the most stable and repeatable outcomes were achieved at 7 min, as shown in Figure 3C.

#### 3.3.4 Template Removing Treatment

To remove the target molecule, the PP-AA@MIP-GCE sensor was immersed various solutions, including AcOH (10 and 15 M), hydrochloric acid (HCl, 1 M), methanol/double-distilled water (MeOH, 1:1, v/v), acetonitrile/double-distilled water (ACN, 1:1, v/v), and acetone for 10 min at 650 rpm and 25 °C on a shaker. The most effective removal of the template molecule was achieved with a 15 M AcOH solution (Figure 3D). The PP-AA@MIP-GCE was immersed in a 15 M AcOH solution and placed on a shaker for varying durations of 5, 7, 10, 12, and 15 min to determine the optimal time for removing the target molecule. The highest peak currents were observed after 10 min (Figure 3E).

#### 3.3.5 Effect of Rebinding Time

The study investigated the incubation time of PP-AA@MIP-GCE with 5.0 × 10^–13^ M VEN on a shaker at 650 rpm and 25 °C for 3, 5, 7, 10, and 12 min. The rebinding time of 5.0 × 10^–13^ M VEN was determined by contrasting the difference (ΔI) in the peak currents (I) after the template molecule was removed and rebonded. The ΔI value increased to a maximum at 7 min and then steadily decreased (Figure 3F). Although the optimal rebinding time of 7 min was short, it did not negatively affect the performance of the sensor. With the prepared sensor surface, it was easily used in both standard solutions and commercial serum samples without damaging the sensor surface. Additionally, the prepared sensor surface was tested using different rebinding solutions on different days. This showed that the prepared sensor achieved stable and reproducible results despite the short rebinding time.

### 3.4 Analytical Performance of PP-AA@MIP-GCE

**Figure 4:**
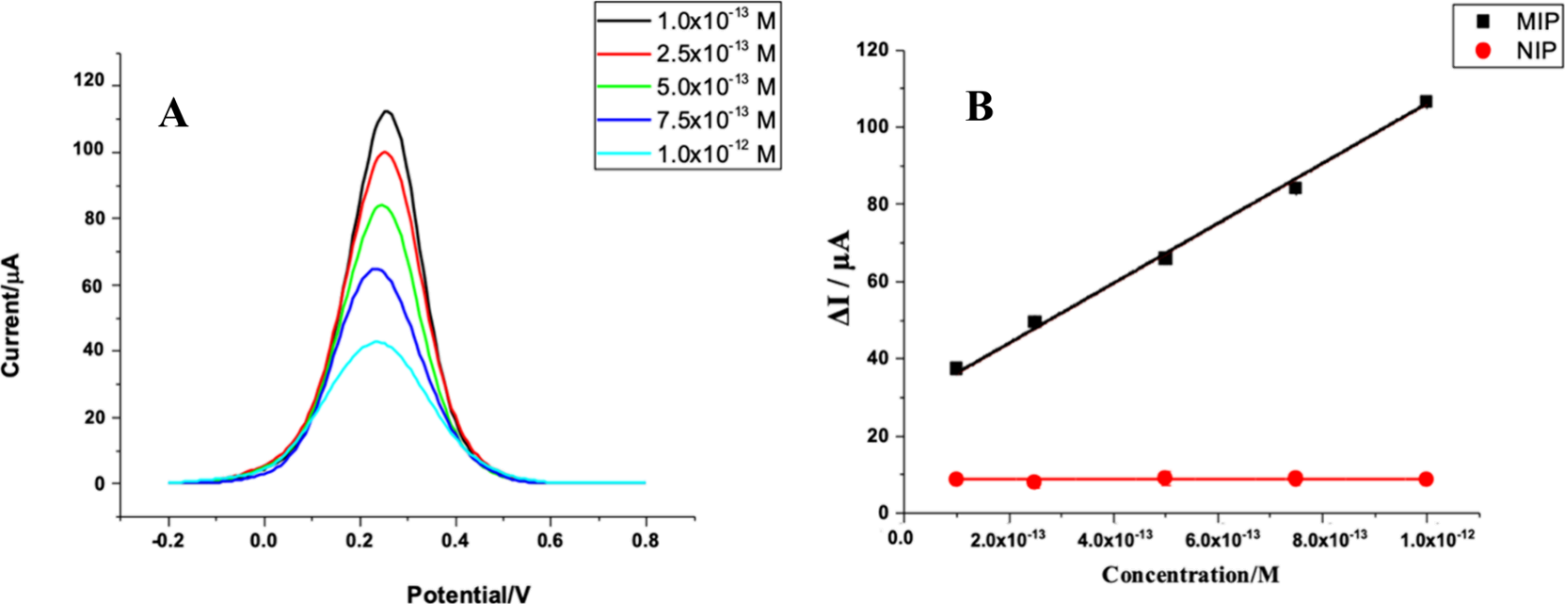
DP voltammograms obtained with different concentrations of VEN rebinding (A) and ΔI values and concentrations of VEN in PP-AA@MIP-GCE and PP-AA@NIP-GCE (B) in standard solution.

The analytical performance of the PP-AA@MIP-GCE sensor was estimated employing DPV under controlled conditions. The regression equation for PP-AA@MIP-GCE was y(Δ*I*/μA) = 7.8 × 10^13^ (concentration of VEN/M) + 29.771 (r^2^ = 0.998). As a result of experimental studies, a linear relationship was found between 1.0 pM and 10.0 pM. In this study, it is found that LOD (LOD = 3 standard deviation/slope) was 0.016 pM, and LOQ (LOQ = 10 standard deviation/slope) was 0.055 pM.^18^Figure 4A shows DP voltammograms recorded after removal of the template molecule and after rebinding of VEN with increasing VEN concentration. The correlation between the ΔI value and the VEN concentration is linear. The analytical performance of PP-AA@MIP-GCE was evaluated by applying the same procedures to PP-AA@NIP-GCE. Figure 4B displays the calibration curves for both sensors by plotting ΔI values against VEN concentration. The ΔI values of the MIP-based sensor increase with concentration, while those of the NIP-based sensor remain constant as the concentration increases.^19^ This comparison demonstrates the specificity of the PP-AA@MIP-GCE sensor for VEN.

**Figure 5:**
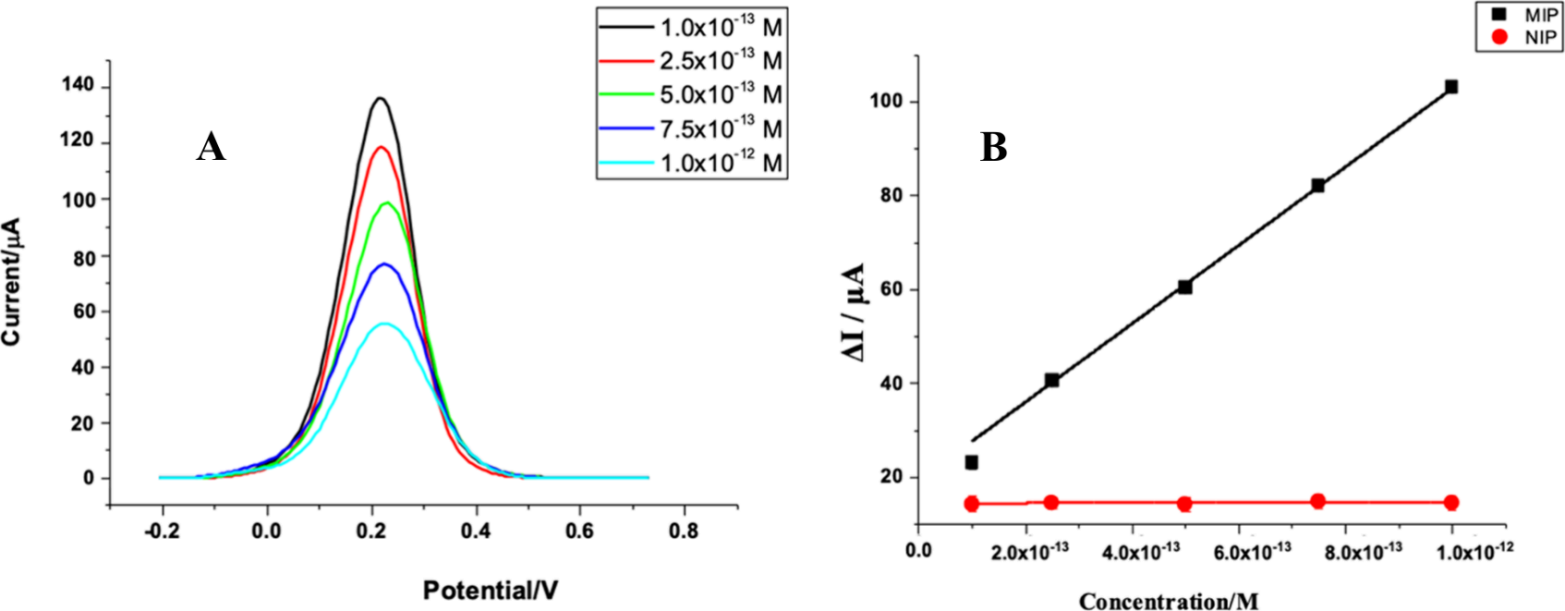
DP voltammograms obtained with different concentrations of VEN rebinding (A) and ΔI values and concentrations of VEN in PP-AA@MIP-GCE and PP-AA@NIP-GCE (B) in the commercial serum sample.

The DPV was utilized to evaluate the detection performance of PP-AA@MIP-GCE in commercial human serum samples. VEN concentrations ranging from 1.0 × 10^–13^ – 1.0 × 10^–12^ M were measured using a 5 mM [Fe(CN)_6_]^3–/4–^ redox probe. The DPV method exhibited a linear correlation between VEN concentration and ΔI values in commercial human serum samples (Figure 5A). Figure 5B compares the calibration curves obtained from the PP-AA@MIP-GCE and PP-AA@NIP-GCE sensors for commercial human serum samples. The plot displays the concentration of VEN versus the corresponding ΔI values.

Precision and accuracy studies were conducted on standard solution and commercial human serum samples using the PP-AA@MIP-GCE sensor. The experiments were conducted using VEN solution at a concentration of 5.0 × 10^–13^ M with five repetitive intraday and interday measurements.^20^ The data obtained from the precision and accuracy studies are presented in Table 1.

**Table 1:** PP-AA@MIP-GCE Sensor’s Validation Results for Standard and Commercial Human Serum Samples

parameters	standard solution	commercial human serum sample
Range of linearity (M)	1.0 × 10^–13^ to 1.0 × 10^–12^	1.0 × 10^–13^ to 1.0 × 10^–12^
Slope (μA/M)	7.76 × 10^13^	8.35 × 10^13^
Standard error of the slope (μA/M)	5.025	4.709
Intercept (μA)	29.771	19163
Standard error of intercept (μA)	0.381	0.262
Determination coefficient (*r*^*2*^)	0.998	0.997
LOD (M)	1.6 × 10^–14^	1.5 × 10^–14^
LOQ (M)	5.5 × 10^–14^	4.9 × 10^–14^
Interday precision (repeatability) (*n* = 5, RSD,^a^ %)	0.452	0.535
Intraday precision (reproducibility) (*n* = 5, RSD, %)	0.920	0.956

a RSD: relative standard deviation.

The stability of the PP-AA@MIP-GCE sensor designed for VEN was tested over a 15-day period, on the first, third, eighth, 10th, and 15th days. The VEN peak current decreased to 99.18% on the third day, followed by further decreases to 89.11% on the eighth day, 83.63% on the tenth day, and 69.28% on the 15th day. The measurements taken on the third day were similar to those taken on the first day, suggesting that the PP-AA@MIP-GCE sensor remained stable for the entire three-day period.

The performance of this sensor was compared to other available methods in the literature in terms of methods, linear range, LOD, LOQ, sample, and recovery % in Table 2. The disadvantages of the literature methods are long preprocessing steps and the use of expensive and toxic materials. On the other hand, the detection limit, linear range, and real sample applications of the fabricated sensor are comparable to the methods in the literature (Table 2). The results show that the proposed method is simple, environmentally friendly, inexpensive, economical, practical, and requires much fewer solvents. Compared with literature methods, this study showed good linearity, reproducibility, reproducibility, low detection limits, selectivity, and stability.

**Table 2:** Comparison of Currently Available Studies in the Literature to This Sensor

method	linear range	LOD	LOQ	sample	recovery (%)	ref
HPLC	0.25–10 μg/mL		0.10 μg/mL	Plasma	>97.2	(1)
RP-HPLC	5.00–0.02 μg/mL	0.075 μg/mL	0.188 μg/mL	Decomposition products	98.66–101.65	(4)
LC-ESI-MS/MS	5.0–5000.0 pg/mL	5 pg/mL	50 pg/mL	Human plasma	90.3–92.5	(14)
RP-HPLC	3–45 μg/mL	0.03 μg/mL	0.0075 μg/mL	Bulk and pharmaceutical samples	98–102	(15)
MIP-based electrochemistry	1.0–10.0 pM (0.868–8.684 pg/mL)	0.016 pM (0.014 pg/mL)	0.055 pM (0.048 pg/mL)	Human serum	99.14–99.20	this work

### 3.5 Specificity Studies

The specificity of the PP-AA@MIP-GCE sensor was evaluated using DPV signals of ibrutinib, piperazine, and azacitidine. This is because the combination of VEN with azacitidine is being studied in phase 1^21^ and with ibrutinib in phase 3.^22^ Additionally, oxidation of VEN produces *N*-oxide on its piperazine moiety.^23^ The specificity of the MIP electrode was appraised by calculating imprinting factors (IF) for PP-AA@MIP-GCE and PP-AA@NIP-GCE.^17^ The ΔI value for each molecule was divided by the value for VEN to determine the imprinting factor (IF). Relative IF (IF’) value is defined as the ratio of IF values for MIP and NIP. An IF’ value greater than 1 indicates high specificity for the target molecule. The structures of molecules and results are presented in Table 3. As predicted, the highest IF value was recorded for VEN. The IF results confirmed the high selectivity of the PP-AA@MIP-GCE sensor for VEN. The results indicate that PP-AA@MIP-GCE has a higher affinity for VEN than other tested molecules.

**Table 3:**
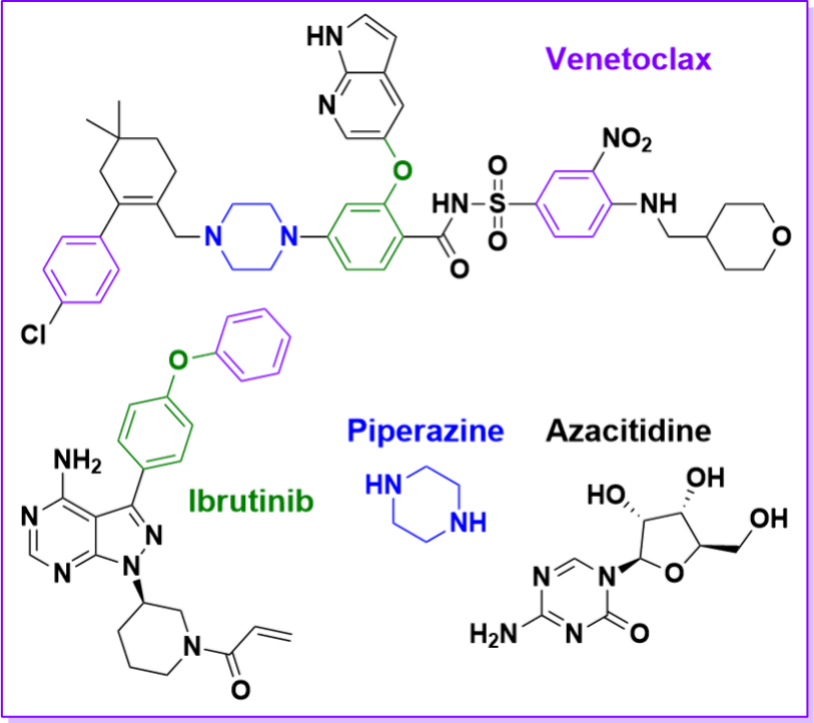
Specificity of PP-AA@MIP-GCE for the Determination of VEN^a^

	MIP		NIP		
molecules	ΔI/μA	IF_(MIP)_	ΔI/μA	IF_(NIP)_	IF′_(MIP/NIP)_
Venetoclax	65.553		15.100		
Ibrutinib	14.780	4.435	11.980	1.260	3.519
Piperazine	13.692	4.788	10.130	1.491	3.212
Azacitidine	12.619	5.195	10.120	1.492	3.482

a Scan rate: 50 mV/s, Potential range: −0.2 V to 0.8 V for DVP method.

### 3.6 Interference Studies

The interference effect of DOP, AA, PAR, UA, Na^+^, SO_4_^2–^, K^+^, and NO_3_^–^ in biological samples was investigated using a PP-AA@MIP-GCE sensor. To determine the impact of interference, VEN was mixed with each component in a 1:10 ratio, and the recovery (%) and RSD (%) values were determined (Table 4).

**Table 4:** Interference Study Results of PP-AA@MIP-GCE Sensor for the Determination of VEN with DPV Method

interfering agent (IA)^a^	recovery (%)	RSD (%)
Dopamine (DOP)	100.61	0.84
Ascorbic acid (AA)	98.65	1.21
Paracetamol (PAR)	100.77	1.29
Uric acid (UA)	98.50	1.34
Na^+^	101.70	0.44
SO_4_^2–^	101.70	0.44
K^+^	100.66	0.63
NO_3_^–^	100.66	0.63

a Molar ratio of VEN/IA is 1:10.

### 3.7 Application of the Sensor

Recovery studies were conducted by adding a standard VEN solution to serum samples at two different known concentrations: 2.50 × 10^–13^ M and 7.50 × 10^–13^ M. Excellent recovery and RSD results were gathered as presented in Table 5.

**Table 5:** Recovery Results of Spiked Commercial Human Serum Samples

	commercial human serum	
parameter	sample #1	sample #2
Spiked amount (M)	2.50 × 10^–13^	7.50 × 10^–13^
Found amount^a^ (M)	2.74 × 10^–13^	7.44 × 10^–13^
RSD (%)	0.34	0.12
Bias (%)	0.86	0.80
Recovery (%)	99.14	99.20

a Mean of five experiments.

## 4 Conclusion

The electrochemical MIP sensor PP-AA@MIP-GCE was developed to determine VEN with remarkable sensitivity and a very low LOD, surpassing the capabilities of most analytical methods used in pharmaceutical analysis. PP-AA@MIP-GCE showed a linear range from 0.1 pM to 1.0 pM for the standard solution, with LOD and LOQ values of 0.016 and 0.055 pM, respectively. The same linear range was observed for a commercial human serum sample, with LOD and LOQ values of 0.015 and 0.049 pM, respectively. The electrochemical detection of VEN in commercial human serum using the selectivity and sensitivity of the MIP electrochemical sensor resulted in a low RSD of 0.34% and a high recovery of 99.14%. This is the first reported use of an electrochemical MIP sensor to determine VEN. The method developed demonstrates promising results for future applications. Additionally, as stated in this report, the remarkable analytical capabilities of the MIP sensor may open new avenues of application beyond developing a highly sensitive electrochemical sensor for VEN and extend its use to the detection of other important compounds. Therefore, the developed sensor, which is affordable, requires no pretreatment and has high selectivity and sensitivity, is promising for routine VEN analysis and could aid in drug monitoring research.

In this study, we have developed the first electrochemical MIP-based sensor to determine VEN, which is simple, inexpensive, and highly sensitive, in contrast to the few previously published analytical methods. The very high selectivity of the PP-AA@MIP-GCE sensor allowed accurate measurement of VEN in human plasma. Moreover, the developed sensor is easy to fabricate and use, reliable, sensitive, highly selective and economical. Thanks to these advantages, PP-AA@MIP-GCE can be considered an analytical tool suitable for disease diagnosis and routine analysis in the pharmaceutical industry. This study is expected to pave the way for future studies on identifying various drug compounds from pharmaceutical preparations, biological samples, and environmental samples, which have high utilization potential.
